# The Structural Complexity and Animal Tissue Distribution of *N*-Glycolylneuraminic Acid (Neu5Gc)-Terminated Glycans. Implications for Their Immunogenicity in Clinical Xenografting

**DOI:** 10.3389/fmolb.2019.00057

**Published:** 2019-07-19

**Authors:** Michael E. Breimer, Jan Holgersson

**Affiliations:** ^1^Department of Surgery, Sahlgrenska Academy, University of Gothenburg, Gothenburg, Sweden; ^2^Laboratory Medicine, Sahlgrenska Academy, University of Gothenburg, Gothenburg, Sweden

**Keywords:** *N*-glycolylneuraminic acid, xenograft, bioprosthetic heart valve, carbohydrate antigen, anti-carbohydrate antibodies, carbohydrate epitope

## Abstract

*N*-Glycolylneuraminic acid (Neu5Gc)-terminated glycans are present in all animal cells/tissues that are already used in the clinic such as bioprosthetic heart valves (BHV) as well as in those that potentially will be xenografted in the future to overcome end stage cell/organ failure. Humans, as a species lack this antigen determinant and can react with an immune response after exposure to Neu5Gc present in these products/cells/tissues. Genetically engineered source animals lacking Neu5Gc has been generated and so has animals that in addition lack the major αGal xenoantigen. The use of cells/tissues/organs from such animals may improve the long-term performance of BHV and allow future xenografting. This review summarizes the present knowledge regarding structural complexity and tissue distribution of Neu5Gc on glycans of cells/tissue/organs already used in the clinic or intended for treatment of end stage organ failure by xenografting. In addition, we briefly discuss the role of anti-Neu5Gc antibodies in the xenorejection process and how knowledge about Neu5Gc structural complexity can be used to design novel diagnostics for anti-Neu5Gc antibody detection.

## Introduction

Products isolated from animal tissues have been used in clinical medicine for a long time as exemplified by porcine insulin introduced in the 1920's and bioprosthetic heart valves (BHV) in 1965 (Binet et al., 1965). In recent years, focus has also been on the potential use in humans of live cells and tissues from animals, primarily pigs, to overcome the shortage of human cells/organs for transplantation (Auchincloss and Sachs, 1998; Cowan and Tector, 2017; Ekser et al., 2017). A major obstacle for transplantation of live animal tissue into humans is the strong xenogeneic immune rejection initiated in the recipient (Auchincloss and Sachs, 1998; Cowan and Tector, 2017; Ekser et al., 2017). The most immediate barrier preventing grafting of porcine tissues into man and non-human primates was shown to be explained by preformed antibodies specific for the Galα1,3Gal (αGal) carbohydrate determinant present on cell surface glycoconjugates (Auchincloss and Sachs, 1998; Ezzelarab et al., 2005). These αGal specific antibodies cause hyperacute rejection of vascularized porcine tissues in humans and non-human primates similar to that caused by preformed anti-blood group ABO antibodies in human allotransplantation (Holgersson et al., 2005). In addition, several non-αGal antigens that humans can develop antibodies against including *N*-glycolylneuraminic acid (Neu5Gc), have been identified and they may contribute to the xeno-rejection process (Ezzelarab et al., 2005; Byrne et al., 2011; Padler-Karavani and Varki, 2011; Galili, 2012; Miyagawa et al., 2012; Salama et al., 2015).

This review summarizes the present knowledge regarding the structural complexity and distribution of Neu5Gc on glycans of BHV as well as cells/organs intended for treatment of end stage organ failure by xenografting. In addition, we discuss how we can use our knowledge regarding Neu5Gc structural complexity for the design of novel diagnostics for anti-Neu5Gc antibody detection. The possible significance of anti-Neu5Gc antibodies in the xenorejection process has been the subject of recent reviews (Padler-Karavani and Varki, 2011; Salama et al., 2015) and will only be commented on briefly in this contribution.

## Chemical Structure Diversity of Sialic Acids Focused on Neu5Gc

**Figure 1:**
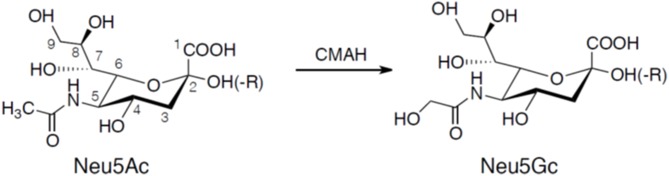
Chemical structures of Neu5Ac and Neu5Gc. Neu5Gc is generated from Neu5Ac by the enzyme CMP-*N*-acetylneuraminic acid hydroxylase (CMAH). Neuraminic acids are linked to the carbohydrate core chain (-R) by a glycosidic linkage involving the hydroxyl group at carbon atom 2 forming an α2-3 or α2-6 linkage. A second neuraminic acid can be added to the penultimate neuraminic acid by an α2-8 linkage.

Sialic acids are α-keto acids with a nine-carbon backbone and are normally placed terminally in the reducing end of glycans (Angata and Varki, 2002; Schauer, 2004). They are found in the deuterostome lineage, i.e., chordates and echinoderms (e.g., sea stars), of animals and in certain bacteria (Angata and Varki, 2002; Schauer, 2004). Sialic acid used to be considered a synonym for *N*-acetylneuraminic acid (5-amino-3,5-dideoxy-D-glycero-D-galacto-2-nonulosonic acid; Neu5Ac), but since its discovery in the 80's the deaminated neuraminic acid, KDN (2-keto-3-deoxy-*D*-glycero-*D*-galacto-nononic acid), is also included in the family of sialic acids (Inoue and Kitajima, 2006). Like *N*-acetylneuraminic acid, KDN is also found in vertebrates and bacteria. The structural diversity among sialic acids is vast with more than 50 distinct molecules that are biosynthetic derivatives of either *N*-acetylneuraminic acid or KDN (Angata and Varki, 2002; Schauer, 2004). *N*-glycolylneuraminic acid (Neu5Gc) is another major type of sialic acid and is also expressed in deuterostomes. The initial characterization of Neu5Gc biosynthesis was explored by Schauer in the 1960's showing that Neu5Ac was converted by CMP-*N*-acetylneuraminic acid hydroxylase (CMAH) to the *N*-glycolyl form by addition of an oxygen atom to the *N*-acetyl group (Schauer et al., 1968; Schauer, 1991) illustrated in Figure 1. Birds, reptiles, amphibians, sperm whales, and several other species including New World monkeys and humans lack CMP-*N*-acetylneuraminic acid hydroxylase and therefore these species lack Neu5Gc (Peri et al., 2017). However, trace amounts of Neu5Gc have been identified in humans, a finding explained by an uptake from ingested meat and dairy products (Schauer et al., 1968; Tangvoranuntakul et al., 2003).

## General Aspects of Glycoconjugates and Anti-carbohydrate Antibodies

### The Structural Diversity of Cell Surface Glycoconjugates

The surface of every cell is covered with a diverse array of glycans, carried by proteins or lipids in the outer plasma membrane leaflet, mediating interactions leading to cell adhesion, trafficking, and signaling (Gustafsson and Holgersson, 2006; Sperandio et al., 2009). Glycans determine self/non-self as they are targets for antibodies of clinical significance in transfusion medicine and transplantation (Holgersson et al., 2005; Gustafsson and Holgersson, 2006). Furthermore, cell surface carbohydrates constitute important attachment sites for viruses, bacteria and bacterial toxins and as such they are required by microbes to initiate infection (Karlsson, 2001; Gustafsson and Holgersson, 2006).

Glycosylation is a common post-translational modification (PTM) of proteins involving enzymatic glycosylation of the protein backbone (Kobata, 2004). The varying sequence and chain length as well as the anomeric configuration (α or β), linkage position and branching sites make glycosylation the structurally most diverse PTM (Dwek, 1995). Covalent modifications of individual sugar residues by sulfation, phosphorylation, acetylation, or methylation add further structural variation to the carbohydrate chain. Therefore, the structural diversity that can be obtained in glycan chains is by far exceeding the complexity obtained by amino acids in polypeptides (Samuelsson and Breimer, 1987).

Two of the most abundant protein glycosylation forms are *N*- and *O*-linked glycosylation. *N*-linked glycans are usually attached via an *N*-acetylglucosamine (GlcNAc) to Asparagine (Asn). They are classified into three types, the high mannose (oligomannose), complex, and hybrid types. *N*-glycan biosynthesis is initiated via the synthesis of the Man_5_GlcNAc_2_ core unit on the dolichol pyrophosphate lipid anchor, which is then re-oriented to the luminal side of the endoplasmic reticulum (ER) membrane and extended to a Glc_3_Man_9_GlcNAc_2_ sequence. Transfer of the Glc_3_Man_9_GlcNAc_2_ oligosaccharide to the consensus sequence (N-X-S/T) in acceptor polypeptides is performed en bloc by the oligosaccharyltransferase (OST). *N*-glycans are further modified in the late ER and Golgi apparatus generating a plethora of *N*-glycan structures. The processing is possibly determined by the function of the glycan structures and the compartment where they are localized, resulting in a species- or even cell type-specific diversity of *N-*linked glycans (Schwarz and Aebi, 2011; Aebi, 2013).

Mucin-type *O*-linked glycans are attached to Ser or Thr via *N*-acetylgalactosamine (GalNAc), but other *O*-glycans may be linked to Ser/Thr via GlcNAc, fucose, glucose, mannose, or xylose (van den Steen et al., 1998). *O*-glycan biosynthesis is initiated in the ER and the chain is further extended in the ER and Golgi by a stepwise addition of monosaccharides. There is no known consensus sequence for initiation of *O*-glycosylation. The initiating step of mucin-type glycosylation is the addition of the GalNAc monosaccharide from UDP-GalNAc to the hydroxyl groups in serine and threonine residues; a reaction catalyzed by a large family of up to 20 different polypeptide GalNAc-transferases (ppGalNAc-Ts) (Bennett et al., 2012). Three distinct regions are recognized in *O*-linked glycans and include the two or three innermost sugar residues nearest the peptide chain constituting the core region, the backbone region contributing to *O*-glycan chain length, and the terminal region with its bioactive determinants (Hanisch, 2001). The determinants are often sialylated, sulfated, acetylated, and/or fucosylated. At least eight different *O*-glycan core chain types, of which cores 1–4 are more common than the rare cores 5–8, have been identified in mammalian glycoproteins. All are based on the innermost αGalNAc residue, which is further substituted at the C3, C6, or both positions (Hanisch, 2001).

Glycolipids are mainly found in the plasma membrane with the lipophilic part (ceramide) integrated in the outer layer of the lipid bilayer and the saccharide chain exposed to the cell environment. In contrast to glycoproteins that carry several different saccharide chains, only one single glycan is attached to each ceramide. As for protein-linked glycoconjugates, glycolipid structural complexity is vast. Immunogenic determinants are linked to various core saccharide chain types (ganglio-, globo-, lacto-, neolacto-series etcetera) (Holgersson et al., 1992). Sialic acid-containing glycolipids (gangliosides) are based on different saccharides of which lactosylceramide and ganglio-series compounds are most abundant.

### Structural Diversity of Neu5Gc-Terminated Glycans

Sialic acids including Neu5Gc are mostly found terminally on glycan chains of glycoproteins and glycolipids. They are commonly linked via an α2,3- or α2,6-linkage to Gal, an α2,6-linkage to GalNAc, or via an α2,8-linkage to another sialic acid (Angata and Varki, 2002; Schauer, 2004). Glycans with the sialic acid linked to other sugar residues and in other binding positions exist (Angata and Varki, 2002; Schauer, 2004). For details regarding the chemical structure of various neuraminic acid-containing glycans, the reader is referred to previously published reviews and text books (Angata and Varki, 2002; Schauer, 2004; Varki et al., 2017).

A variety of Neu5Gc-terminated *N*- and *O*-glycans have been identified. Using CHO-K1 cells as host cells and a mucin-type fusion protein as a reporter protein to study *O*-glycosylation, sialylated core 1, core 2, core 3, and extended core 1 *O*-glycans were identified following transient co-expression of the different core enzymes in CHO-K1 cells (Liu et al., 2015). Between 5 and 10% of the sialylated *O*-glycans carried Neu5Gc and it was found α2,3- and α2,6-linked (following expression of ST6Gal I) to Gal and α2,6-linked to GalNAc (Liu et al., 2015). Choi and co-workers used matrix-assisted laser desorption/ionization time-of-flight mass spectrometry (MALDI-TOF MS) to study *N*-glycans released from native porcine heart valves or heart valves treated with α-galactosidase (Choi et al., 2012). They identified a number of complex type *N*-glycans carrying Neu5Gc (Choi et al., 2012). The full extent of the structural diversity of *N*- and *O*-glycans carrying Neu5Gc remains to be elucidated. However, a not too brave assumption is that the majority of glycans carrying Neu5Ac have their Neu5Gc counterpart.

The most common Neu5Gc-terminated glycolipid is the GM3 ganglioside with Neu5Gc linked to lactosylceramide (Iwamori and Nagai, 1978; Gasa and Makita, 1980; Hanagata et al., 1990). Complex Neu5Gc-containing gangliosides with several sialic acids have been identified (Ohashi and Yamakawa, 1977; Ariga et al., 1983; Nakao et al., 1991), also in various combinations with blood group ABO and Lewis antigen determinants (van Dessel et al., 1979; Nohara et al., 1990). Terminal sialic acid disaccharides exist in all the possible combinations NeuGc-NeuGc-, NeuAc-NeuAc-, NeuGc-NeuAc-, and NeuAc-NeuGc- (Watarai et al., 1991).

### Recognition of Saccharide Structures by Anti-carbohydrate Antibodies

Traditionally, carbohydrates have been considered T lymphocyte-independent antigens because they activate B lymphocytes without T-cell help. As most carbohydrates cannot be presented via MHC class II antigens and, thus, not recruit T-cell help, the B-cell response lack affinity maturation and is skewed toward the production of IgM and IgG2 antibodies in human (Vos et al., 2000). To overcome the lack of T-cell help in the response of B-cells to carbohydrate antigens, neoglycoconjugates have been developed by coupling the carbohydrate antigen to carrier proteins. Upon intracellular processing, peptides from the latter can be presented by MHC class II antigens on B-cells to T-cells that upon activation can provide help to the B-cell. A good example of this is the *Haemophilus influenzae* neoglycoconjugate vaccine (Micoli et al., 2018). Polysaccharides carrying both negatively and positively charged substituents have been shown to interact with MHC class II species (Avci and Kasper, 2010), as have oxidative breakdown products of polysaccharides (Velez et al., 2009). Anti-carbohydrate antibodies are normally of low affinity, often of 10^3^-10^5^ times less affinity than anti-peptide or -protein antibodies (Krause and Coligan, 1979; MacKenzie et al., 1996; Brorson et al., 2002). The low affinity is compensated for by a high avidity provided for by the decavalent configuration of the IgM antibody or self-associated IgG2 antibodies in humans (Greenspan et al., 1988; Cooper et al., 1991). Multivalently configured, as in IgM or self-assembled IgG2, anti-carbohydrate antibodies facilitate high avidity binding to multivalently expressed or clustered carbohydrate antigens on the surface of cells, bacteria, and viruses. They are thus ideally suited to distinguish cells expressing high densities of a carbohydrate antigen from those expressing low densities of the same antigen.

The low affinity of anti-carbohydrate antibodies (and lectins) as opposed to anti-peptide antibodies may be explained by the contribution of entropic factors to binding, which is not solely reliant on enthalpic factors (reviewed in Haji-Ghassemi et al., 2015). Because of the flexible nature of carbohydrates, antibody binding requires unfavorable immobilization of otherwise flexible parts of the saccharide chain and, thus, loss of entropy (Haji-Ghassemi et al., 2015). Therefore, extension of the sugar chain and fixation of the anomeric carbon in one conformation may increase antibody binding affinity even if the extending sugar is not involved in the binding (Haji-Ghassemi et al., 2015). Further, the entropic consequences of water in binding of anti-carbohydrate antibodies are hard to predict because solvating water molecules may need to be displaced or trapped during antibody-antigen complex formation (Haji-Ghassemi et al., 2015).

Early studies on the structural features of anti-carbohydrate antibodies suggested that the antibody binding site could encompass up to six monosaccharide residues and to be pocket- or groove-shaped (Kabat, 1978). Pocket-shaped for binding determinants placed terminally in the saccharide chain and groove-shaped for binding internally on polysaccharide structures. In their comprehensive review, Haji-Ghassemi and co-authors concluded after reviewing the structural features of anti-carbohydrate antibodies specific for over 20 antigens, that even though they share characteristic features there are no general rules governing their behavior (Haji-Ghassemi et al., 2015).

The crystal structure of the Fab fragment of the murine anti-Neu5Gc antibody has been resolved at a 2.2 Å resolution and a molecular model of this fragment in complex with the saccharide moiety of *N*-glycolyl GM3 ganglioside has been generated (Krengel et al., 2004; Bjerregaard-Andersen et al., 2018).

## Structural Complexity and Species/Tissue Distribution of Neu5Gc in Tissues of Relevance for Bioprosthetic Heart Valves

Several types of bio-devices of animal origin have been developed for clinical use. Examples of these are sheets to build up the abdominal wall in the repair of hernias (Patel et al., 2018) and BHV to replace diseased heart valves (Fiedler and Tolis, 2018). BHV used clinically are mainly produced from bovine, porcine, and equine tissues such as pericardium and heart valves. The tissues are processed, encompassing for example glutaraldehyde, ethanol, and anti-calcification, to reduce immunogenicity and to extend preservation times of the tissues. Carbohydrates are resistant to many of these treatments as shown by remaining αGal antigens in commercial BHV products (Kasimir et al., 2005; Naso et al., 2013). Sialic acids are negatively charged (“acidic” carbohydrate components) and are slightly more sensitive to chemical degradation compared to neutral saccharide components. However, sialic acid-terminated saccharides have been identified by immunohistochemistry in formaldehyde-fixed tissue sections (Morozumi et al., 1999; Magnusson et al., 2005) and a recent study did not find any change in anti-Neu5Gc staining of naïve and glutaraldehyde-treated (0.02–2%) porcine valves indicating that these saccharides may resist the processing treatments (Lee et al., 2016). However, BHVs available for clinical use contain extremely small amounts of biological tissue and are very expensive, why it is difficult to perform structural investigations on antigen expression using chemical methods. Therefore, studies on native animal pericardium and heart valve tissues have been performed to make a chemical characterization possible. Bearing in mind that carbohydrate determinants, at least in part, remain intact despite the processing of the tissue.

### Valve Cusps

Immunohistochemical analysis of naïve porcine aortic valve cusps showed a strong Neu5Gc staining of the cusp endothelium (Reuven et al., 2016). Using immunohistochemistry, Lee and coworkers tested pig heart valves obtained from wild-type, GTKO/CD46 and GTKO/CD46/NeuGcKO animals and a strong Neu5Gc expression was found in wild-type and GTKO/CD46 tissues that was absent in the GTKO/CD46/NeuGcKO valves (Lee et al., 2016).

Terminal Neu5Gc saccharides (assumed to be the Hanganutziu-Deicher, HD, antigens) have been identified by mass spectrometry in *O*-glycans isolated from naïve pig aortic and pulmonary valves (Jeong et al., 2013). A more complex pattern of Neu5Gc-terminated saccharides was found in the aortic valves compared to the pulmonary valves and the heart muscle.

In investigations of glycolipids of naïve animal heart valves and pericardia, an unexpected finding was the lack of Neu5Gc-terminated gangliosides in pig heart valves (Barone et al., 2014), while the pig, bovine, and equine pericardia all contained gangliosides with terminal Neu5Gc residues (Barone et al., 2018). Neu5Gc-GM3 was found in all animal species while other gangliosides showed a species-specific distribution; Neu5Gc-GD3 (equine), Neu5Gc-GM1 (pig, bovine), Fuc-Neu5Gc-GM1 (pig). These structures were deduced by a combination of thin-layer chromatographic mobility, staining by the HD antigen-specific chicken monoclonal antibody (HU/Ch2-7; Asaoka et al., 1992) in combination with liquid chromatography-mass spectrometry of purified ganglioside fractions (Barone et al., 2018).

### Pericardium

Immunohistochemical analysis of naïve porcine and bovine pericardia showed anti-Neu5Gc staining of the matrix of the pericardium as well as the endothelium of a small artery and a capillary (Reuven et al., 2016). A strong Neu5Gc expression was found in wild-type and GTKO/CD46 pig pericardium while pericardia from GTKO/CD46/NeuGcKO animals were negative (Lee et al., 2016).

### Studies on BHV Used in the Clinic

The commercial BHVs used in the clinic are mainly produced from bovine pericardia even if some manufacturers use porcine valves and porcine as well as equine pericardia (Reuven et al., 2016). Immunostaining and HPLC analysis of homogenates from six different commercial BHV revealed presence of Neu5Gc in all products but the limited amount of tissue did not allow any further exploration of saccharide structures (Reuven et al., 2016). In another study, three different commercial BHV valves were tested and all showed strong anti-Neu5Gc binding as well as binding of human serum (Lee et al., 2016).

## Structural Complexity and Species/Tissue Distribution of Neu5Gc in Tissues of Relevance for Xenotransplantation

### Endothelial Cells

Flow cytometric analysis using the HU/Ch2-7 antibody specific for HD antigens revealed strong expression of HD antigens in cultures of porcine and bovine aortic endothelial cells and immunohistochemical analysis of porcine kidney revealed strong expression in all vascular endothelial cells (Morozumi et al., 1999; Reuven et al., 2016). Also, pericardial vessel endothelium contained Neu5Gc glycans (Reuven et al., 2016).

Bouhours and co-authors studied gangliosides from primary cultures of porcine endothelial cells labeled with ^14^C-monosaccharides and were able to identify the GM3 and GD3 compounds with *N*-glycolylneuraminic acid as their predominant sialic acid (Bouhours et al., 1996).

Even if not all animal organs corresponding to the vascularized organs currently used in clinical transplantation have been analyzed for Neu5Gc expression in the specific organ, it can be anticipated that endothelial cells of these organs express Neu5Gc as shown for pig kidney endothelium (Reuven et al., 2016).

### Pancreatic Islets

Glycoproteins carrying *N*-linked HD determinants have been identified in adult pig islet cells together with several other sialic acid-capped compounds that reacted with human natural antibodies (Komoda et al., 2004). In addition, porcine pancreas was shown to contain gangliosides with Neu5Gc (Nakamura et al., 1984).

### Cornea

Our knowledge regarding corneal xenotransplantation has increased considerably and corneal grafting is, together with pancreatic islets, close to be tested in human clinical trials. Neu5Gc have been identified by immunohistochemistry in all layers of pig cornea (Cohen et al., 2014). Mass spectrometric analyses of pig corneal endothelial cells and keratocytes releveled several *N*-glycans with terminal Neu5Gc (Kim et al., 2009). Because cornea is a non-vascularized tissue, the clinical relevance of Neu5Gc antigen expression in this tissue remains to be elucidated.

### Lymphocytes

During reperfusion of grafted vascularized organs, considerable amounts of blood cells, including leukocytes, trapped in the organ are transferred to the recipient and may induce an immune response. Leukocytes remain in the harvested organs despite extensive rinsing of the vascular tree with perfusion solution (Magnusson et al., 2003). Therefore, knowledge regarding carbohydrate antigen expression also in lymphocytes is of importance.

Porcine spleen lymphocytes contain a complex ganglioside mixture with Neu5Gc-GM3 and -GD3 as major constituents (Hueso et al., 1985), while the ganglioside mixture of peripheral blood lymphocytes was less complex with Neu5Gc-GD3 as the major ganglioside species (Hueso et al., 1985; Magnusson et al., 2003).

Studies on peripheral blood lymphocytes and thymocytes of calves revealed GM3 as major component and that 97% of the gangliosides from peripheral cells contained Neu5Gc, while the ganglioside composition of thymic cells was more complex containing several ganglioside species including Neu5Ac sialic acids (Dyatlovitskaya et al., 1980).

### Vascularized Organs

Most studies identifying Neu5Gc antigens in animals have been performed on mouse, bovine, rabbit, and sheep tissues. Studies on vascularized organs of pigs, the most likely species to be used for xenografting, are limited. However, Neu5Gc-containing gangliosides have been structurally characterized in porcine plasma (Hanagata et al., 1990), skeletal muscle (Ariga et al., 1983), adipose tissue (Ohashi and Yamakawa, 1977), peripheral nerve (Magnusson et al., 2005), small intestine (Diswall et al., 2007, 2014), kidney (Diswall et al., 2007), and pancreas (Nakamura et al., 1984; Diswall et al., 2007), and it can therefore be anticipated that Neu5Gc-terminated glycans are present in all porcine organs. Perhaps with the exception of the brain where Neu5Gc appears to be sparsely expressed (Davies and Varki, 2015).

Neu5Gc linked to GalNAc on *O*-glycans has been identified in pig heart muscle (Jeong et al., 2013). Studies using the anti-HD antibody revealed Neu5Gc-terminating glycolipid compounds in pig hearts and Neu5Gc-GM3 was the most abundant one (Diswall et al., 2010). Several more complex ganglioside species were found but not structurally characterized in detail.

Pig kidneys show strong anti-Neu5Gc staining of all vascular endothelial cells and brush border tubular cells, while the smooth muscle cells of arteries are negative (Reuven et al., 2016). Like the situation in the heart, Neu5Gc-terminating glycolipids were identified in pig kidneys by the anti-HD antibody and Neu5Gc-GM3 was the most abundant one (Diswall et al., 2010). *N*-glycans released from pig kidney cell membrane glycoproteins revealed several novel Neu5Gc-terminated saccharides with up to 14 monosaccharide units present in complex branched structures (Kim et al., 2008). These studies were performed by a combination of HPLC separation of released saccharides followed by MALDI-TOF mass spectrometry. Monosaccharide residues were identified by exoglycosidase digestion (Kim et al., 2008).

## Anti-Neu5Gc Antibodies With Special Reference to Induced Anti-Neu5Gc Antibodies in Humans Exposed to Animal Tissue

Hanganutiziu and Deicher (HD) antibodies, the immunodominant group of which is Neu5Gc, were originally identified based on their ability to agglutinate erythrocytes of many animal species (Hanganutziu, 1924; Deicher, 1926). HD antigen-active molecules were later isolated from equine and bovine erythrocytes and were shown to include the Neu5Gc-LacCer (Neu5Gc-GM3) and Neu5Gc-nLc_4_Cer glycosphingolipids (Naiki and Higashi, 1980; Mukuria et al., 1986a,b). A glycoprotein from bovine erythrocytes was also shown to be HD antibody-reactive (Naiki and Higashi, 1980; Mukuria et al., 1986a,b). Anti-Neu5Gc antibodies, then defined as HD antibodies, were originally found in sera of patients injected with animal serum but has since then been identified in patients with various malignancies (Malykh et al., 2001) and chronic inflammatory diseases (Padler-Karavani et al., 2013). Whether or not anti-Neu5Gc antibodies are present in the serum of healthy individuals is debated and contradicting results exist in the literature (Mukuria et al., 1986b; Kobayashi et al., 2000; Tangvoranuntakul et al., 2003; Nguyen et al., 2005; Padler-Karavani et al., 2008; Blixt et al., 2009; Huflejt et al., 2009; Le Berre et al., 2017; Leviatan Ben-Arye et al., 2019). To some extent, but perhaps not fully, can these discrepant results be explained by differences in assays and substrates used for their detection (Mukuria et al., 1986b; Kobayashi et al., 2000; Tangvoranuntakul et al., 2003; Nguyen et al., 2005; Padler-Karavani et al., 2008; Blixt et al., 2009; Huflejt et al., 2009). Like blood group ABO (Holgersson et al., 2014), sialyl-Lewis x (Lofling and Holgersson, 2009), and anti-αGal antibodies (McKane et al., 1998) recognize their determinants in a structural context-dependent manner, so do anti-Neu5Gc antibodies (Padler-Karavani et al., 2008). Thus, to detect all Neu5Gc antibodies and not to miss a part of the anti-Neu5Gc repertoire, it is important that the assays used are based on a broad repertoire of Neu5Gc-terminated glycans linked to different core chains and with different linkage configurations between Neu5Gc and the penultimate sugar residue (Padler-Karavani et al., 2008). For this purpose, the glycan microarray and in which antibody reactivity with pairs of Neu5Ac- and Neu5Gc-terminated glycans based on the same core saccharide chain are compared, appears optimal as the differential and preferred reactivity with the Neu5Gc glycan can be directly ascribed to the *N*-glycolyl group (Padler-Karavani et al., 2011, 2012; Leviatan Ben-Arye et al., 2017, 2019; Bashir et al., 2019).

Like other anti-carbohydrate antibodies, anti-Neu5Gc antibodies develop during the first year of life. However, in contrast to for example ABO antibodies that are believed to be induced in response to bacteria carrying A- or B-like determinants in their lipopolysaccharide or capsular polysaccharide, it is hypothesized that anti-Neu5Gc antibodies are induced by commensal/pathogenic, non-typeable *Haemophilus influenzae* which have taken up Neu5Gc from the diet and incorporated it into its cell surface lipooligosaccharide (Taylor et al., 2010). When it comes to the induced immune response to Neu5Gc following, for example grafting of animal cells/tissues or administration of animal/recombinant proteins carrying Neu5Gc-glycans our knowledge is limited.

Immunization of renal allotransplant recipients upon rabbit anti-human thymocyte induction therapy showed an IgG antibody response with an expanded diversity and *de novo* recognition of different anti-Neu5Gc glycans (Amon et al., 2017). Exposure of humans to anti-thymocyte globulin was associated with a shift in the anti-Neu5Gc IgG repertoire and affected the outcome of subsequent renal allografts (Mai et al., 2018). However, repeated injections of recombinant human erythropoietin produced by Chinese hamster ovary cells expressing 1% Neu5Gc did not result in any significant production of anti-Neu5Gc-specific antibodies (Noguchi et al., 1996).

Kobayashi and co-workers studied the anti-Neu5Gc antibody response in patients grafted with fetal porcine pancreatic islets (Groth et al., 1994) and in patients who had their circulation connected to a pig kidney *ex vivo* (Breimer et al., 1996; Rydberg et al., 1996). No significant elevation of IgG and IgM antibody levels against the Neu5Gc-GM3 ganglioside was observed in sera from these patients (Kobayashi et al., 2000). However, the Neu5Gc-GM3 coated ELISA used in this study was later found to be sufficiently sensitive. When individual patients from these clinical trials were tested using a glycan microarray an increase of anti-Neu5Gc antibodies was found in some patients transplanted with pig islets (Blixt et al., 2009). In one of the two patients, who had their circulation connected to a pig kidney, an increase in antibodies binding to Neu5Gc-terminated GM3 and GD3 gangliosides isolated from pig kidney was found (Magnusson et al., 2003).

Studies of burn patients exposed to live pig skin revealed a statistically significant increase in serum levels of anti-Neu5Gc antibodies in patients compared to controls (Scobie et al., 2013). However, the increase in the mean anti-non-αGal IgG antibody level in the patient group was due to some patients responding, while other patients did not show any increase in anti-non-αGal IgG antibody levels. Blocking studies in selected patients, using Neu5Gc/Neu5Ac, suggested that Neu5Gc glycans were the major non-αGal antigens that induced the antibody response, although other non-αGal antigens might also be involved (Scobie et al., 2013).

Studies on xeno-antibody responses in patients grafted with BHV have been conducted focusing on anti-Gal antibody levels, which were shown to be increased in patients receiving BHVs compared to controls (Konakci et al., 2005; Bloch et al., 2011; Park et al., 2013). So far, no studies investigating anti-Neu5Gc antibody levels following BHV implantation have, to our knowledge, been reported.

In summary, the knowledge regarding the immune response to Neu5Gc glycans in humans exposed to animal tissues is limited as is the knowledge regarding the potential clinical significance of anti-Neu5Gc antibodies in allo- and xenograft rejection.

## Novel Anti-Neu5Gc Antibody Diagnostics Should Detect as Much as Possible of the Diverse Anti-Neu5Gc Antibody Repertoire in a High-Throughput and Reproducible Manner

In 1986, Mukuria et al. described an enzyme-linked immunosorbent assay (ELISA) for detection of HD antibodies using flat-bottomed 96-well plates coated with purified Neu5Gc-LacCer (Mukuria et al., 1986b). There was an overall good correlation between the HD antibody reactivity obtained with the ELISA and the horse erythrocyte hemagglutination (HA) test (Mukuria et al., 1986b). However, ~3% of the sera were negative in the ELISA despite a positive HA suggesting that some anti-Neu5Gc antibodies were not detected in the ELISA (Mukuria et al., 1986b). Using another ELISA format in which polyacrylamide (PAA)-based neoglycoconjugates carrying a single Neu5Gc residue in multiple copies were coated in the wells, most human sera were shown to contain anti-Neu5Gc antibodies (Tangvoranuntakul et al., 2003). Reactivity with the corresponding Neu5Ac-PAA glycoconjugate was used as background control. Using flow cytometry and α-galactosidase-treated porcine RBC as target cells in the absence and presence of 7.5 mM Neu5Gc, 17/20 sera from healthy volunteers were shown to contain anti-Neu5Gc antibodies (Zhu and Hurst, 2002).

Realizing that the anti-Neu5Gc repertoire, like other anti-carbohydrate antibody repertoires, is polyclonal and binds Neu5Gc in different structural contexts determined by the underlying carbohydrate core chain, Padler-Karavani and coworkers developed a novel, innovative ELISA inhibition assay (EIA) aimed at detecting and quantifying a broader portion of the anti-Neu5Gc repertoire (Padler-Karavani et al., 2013). The EIA relied on the difference in reactivity of anti-Neu5Gc antibodies in human serum with WT and *Cmah*-KO mouse serum (Padler-Karavani et al., 2013). To remove all human antibodies reacting with mouse protein and carbohydrate antigens except Neu5Gc, human serum was pre-absorbed on mouse serum from *Cmah*-KO mice and then incubated in wells coated with mouse serum from WT mice. The rationale being that only remaining anti-Neu5Gc antibodies are detected on WT mouse serum. Using this assay, the authors detected an elevated anti-Neu5Gc response in patients with an acute Kawasaki's disease compared to patients with aneurysms or dilated coronary arteries (Padler-Karavani et al., 2013). A potential caveat with this assay is the fact that the proteome and glycome of mouse serum may vary between individuals of the same strain and between the *Cmah*-KO and WT strains even though they are of the same genetic background. Thus, reproducibility over time can be hard to achieve.

Printed glycan microarrays are powerful tools for determining the fine binding specificity of glycan-binding proteins such as carbohydrate-specific antibodies (reviewed in Smith et al., 2010; Rillahan and Paulson, 2011). Arrays directed at determining the fine specificity of sialoside-binding proteins have been developed (Padler-Karavani et al., 2011, 2012; Leviatan Ben-Arye et al., 2017). They have been successfully used to determine the fine specificity of sialoside-binding plant and animal lectins as well as carbohydrate-binding antibodies (Padler-Karavani et al., 2011, 2012; Leviatan Ben-Arye et al., 2017). By printing pairs of Neu5Ac- and Neu5Gc-terminated glycans, the specificity of polyclonal and monoclonal anti-Neu5Gc antibodies have been elucidated (Leviatan Ben-Arye et al., 2017). A high-throughput format of the latter can be used to assess 16 serum samples on one printed slide (Leviatan Ben-Arye et al., 2017). It is important, however, to realize that the chemistries used to produce, present and couple the glycan to the glass slide will all influence the results. Thus, glycan arrays carrying identical glycan structures may not always give similar results (Padler-Karavani et al., 2012; Bashir et al., 2019).

Despite, the very important contributions of glycan arrays to the specificity-determination of anti-Neu5Gc antibody repertoires in health and disease, there is still a need for novel assays allowing quantification of the structurally diverse anti-Neu5Gc repertoire in a reproducible manner and which can be used in clinical routine laboratories on large patient cohorts. Investigations of large patient cohorts suffering from various chronic inflammatory and malignant disorders will be necessary to investigate the full scope of the medical importance of anti-Neu5Gc antibodies.

## Conclusion

In addition to the αGal antigen determinant, glycans with terminal Neu5Gc residues may constitute an immunogenic barrier for xenografts into humans. However, firm evidence for the role of Neu5Gc antibodies in xenograft rejection is lacking. Because the immune biology of the anti-Neu5Gc response is slightly different from both the ABO and anti-Gal antibody responses, further studies are needed to better define the exact role of the Neu5Gc antibody repertoire in the xenorejection process. Because carbohydrate antigens are quite resistant to destruction/removal by the procedures used in the manufacturing of bioprosthetic products of animal origin, these antigen determinants must be considered when using live as well as chemically modified animal cells/tissues/organs for treatment of end stage human organ failure. Animals genetically engineered to silence the CMP-*N*-acetylneuraminic acid hydroxylase (CMAH) responsible for the biosynthesis of Neu5Gc have been generated and may be used as source animals for future xenografting including procurement of tissues for bio-prosthesis manufacturing.

## Author Contributions

All authors listed have made a substantial, direct and intellectual contribution to the work, and approved it for publication.

### Conflict of Interest Statement

The authors declare that the research was conducted in the absence of any commercial or financial relationships that could be construed as a potential conflict of interest.
